# Pulse distortion caused by waveguide inhomogeneity in nonlinear optical wavelength converters

**DOI:** 10.1038/s41598-023-28799-3

**Published:** 2023-01-27

**Authors:** Danial Paygan, Mohammad Amin Izadi, S. Faezeh Mousavi, Rahman Nouroozi

**Affiliations:** 1 Institute for Advanced Studies in Basic Sciences (IASBS), 444 Prof. Yousef Sobouti Blvd., Zanjan, 45137-66731 Iran; 2 Department of Physics, University of Trieste, 34127 Trieste, Italy; 3 CNR-INO, National Institute of Optics, Area Science Park, 34149 Basovizza, TS Italy

**Keywords:** Applied physics, Electronics, photonics and device physics, Photonic devices

## Abstract

Low-noise integrated all-optical wavelength converters that can be operated in short pulse regime are essential tools to overcome contention resolution in a modern communication network, based on wavelength division multiplexing. Any imperfect functionality in such devices causes non-ideal optical power transfer to the converted data pulses. All imperfections during the preparation and operation of the wavelength converters can be addressed to the waveguide inhomogeneity which distorts data pulses to be converted. This paper reports different waveguide inhomogeneity effects on the pulse distortion while using periodically poled lithium niobate waveguide as wavelength converters. Three types of $\chi ^{(2)}$-based nonlinear optical processes, including second harmonic generation, difference frequency generation, and cascaded second harmonic generation/difference frequency generation are numerically studied to show that any constant, linear, and quadratic waveguide inhomogeneity causes short pulse (down to 1 ns) distortion in such wavelength converters. In addition, it is shown that the reconstruction of $\textrm{sech}^2$-shaped generated pulses is possible, when suitable upside-down quadratic variations of obtained inhomogeneity are deliberately induced in the waveguide. Notably, for pulsed second harmonic generation, the generated pulse can be compressed using an upside-down quadratic phase mismatch.

## Introduction

Wavelength division multiplexing (WDM) is an effective technique to increase the transmission capacity of telecommunication systems. In a WDM system, communication bandwidth is occupied by multiple, independent, and spectrally distinct wavelength channels carrying encoded data in advanced modulation formats such as phase, time, and polarization^1,2^. Principally, WDM routes data channels based on desired wavelengths from the transmitter to the related receiver in a wavelength-selective network. Therefore, the channels are restricted by allocating the same wavelength throughout the path. Wavelength-selection constraints may cause channel blocking between the nodes and interrupt the communication network. Hence, the wavelength converter plays the role of a low-loss executable solution against wavelength contentions. It changes the wavelength of input data-carrying waves into shifted unoccupied ones^3^.

Practically, high-speed all-optical manipulation of encoded data in conventional optical channels can be achieved by electro-optical and/or nonlinear effects in integrated optically-based devices. Wavelength, phase, amplitude, and polarization of the electric field for an optical communication signal can be modulated using integrated optical devices in Lithium Niobate (LN) substrate. The outstanding properties of LN can be listed as (I) ultra-fast optical response, (II) high transparency over a wide range of frequencies from ultra-violet to far infra-red, (III) excellent waveguide properties fabricated via Ti in-diffusion or proton exchange methods, (IV) the coherent and efficient transformation of encoded data with a large second-order nonlinear coefficient ($d_{33}$), and (V) its ferroelectricity behavior^4–6^. The last factor is essential to perform Periodically Poled Lithium Niobate (PPLN) gratings where the phase mismatch between interacting waves in the nonlinear optical process can be removed effectively via quasi phase matching (QPM) technique^7,8^. Using the quasi-phase matched PPLN waveguides with different spatial periods, wavelength conversions within the whole transparency range of LN are achievable. Considering the impressive role of QPM in the efficient wavelength converters via PPLN, the efficiency improvement of this technique has been investigated theoretically and experimentally^9–11^, after its first demonstration by Armstrong ^7^ in 1961. In a PPLN, the periodic pattern of $\chi ^{(2)}$ direction leads to the formation of a nonlinear grating where its pitch is $\frac{2\pi }{\Delta \beta }$. Here, $\Delta \beta$ is the phase mismatch between the interacting waveguide modes and will be introduced in the next section.

It is convenient to consider homogeneous preparation of waveguides and desired QPM gratings. However, many factors cause their imperfect formation which leads to non-efficient wavelength conversion in both continuous wave (CW) and pulsed regimes. Among such factors, the thermal effects during the fabrication process ^11^ and the penetration depth of the induced static electric field for grating formation are the main obstacles to achieve appropriate homogeneity in PPLN waveguides. Hitherto, there have been some works for consideration of inhomogeneities in optical waveguides. For instance, Sodha and Ghatak had a comprehensive survey in the effects of inhomogeneity on the general properties of optical waveguides, ignoring its effects on the pulse propagation^12^. Moreover, in a practicability point of view, a numerical method for determining the propagation characteristics of inhomogeneous planar optical waveguides was proposed, just with consideration of linear functions and no wavelength conversions^13^. An accurate study of the inhomogeneity along or across the optical waveguides was also presented, without considering the effects on nonlinear processes^14^. In another study, the effects of 3-dimensional scatterers on the propagating optical waves were examined, too^15^. In addition, the effects of inhomogeneities on nonlinear integrated optical components were studied by Janet Jackel^16^ where the main focus was on the relative sensitivity of interferometers and directional couplers. Furthermore, the current fabrication limits and their impact on the final performance of Ti:LiNbO3 waveguides was studied, and the link between fabrication errors of waveguides and their final performance was discussed. That work implied that the imperfections are important for quantum state generation and manipulation, without any discussion on the pulse propagation which stands in classical photonics^17^. Since the effects of nonlinear inhomogeneities on the pulse propagation is absent in all mentioned references, a specific type of waveguide inhomogeneity in a cascaded second harmonic generation/difference frequency generation (cSHG/DFG) has been studied in Ref. ^11^. However, many different types of imperfections can be occurred during the formation and operation of PPLN optical waveguides. Therefore, having a comprehensive vision of the waveguide inhomogeneity effect on the pulsed wavelength conversion performance is crucial in nonlinear photonics.

In an ideal (homogeneous waveguide geometry and symmetric duty cycle of the PPLN grating), the quasi-phase matching condition of the process should be fulfilled. Any deviation from such condition results in a phase mismatch along the waveguide and efficiency reduction of the process. Such a phase mismatch results non ideal power transfer from the pump to the generated modes and distorts the transmitted pulses. In this way, an overall estimation of PPLN waveguide inhomogeneity can be achieved.

This article reports the numerical study of the pulsed wavelength conversion in presence of different induced inhomogeneities for PPLN waveguides. The inhomogeneities are considered to be categorized in various functions (constant, linear, quadratic, and upside-down quadratic) of phase mismatches ($\Delta \beta$). It should be noted that, up to a best approximation, the formation of nonlinear gratings for quasi phase matching techniques, does not affect the linear optical properties of the waveguide. Due to this reason, the main focus of this paper is on the nonlinear phase mismatch distributions and their useful applications. Hence, the effects of such inhomogeneities are investigated in three conventional nonlinear interactions in wavelength converters: second harmonic generation (SHG), difference frequency generation (DFG), and cSHG/DFG. The well-known split step Fourier transform (SSFT) method is used ^18,19^ for such investigation. Accordingly, the rest of this paper is as follows: in the section of “Methods and results”, the CW and pulsed SHG, DFG, and cSHG/DFG processes will be examined for different inhomogeneous PPLN waveguides (constant, linear, quadratic, and upside-down quadratic variations of $\Delta \beta$). In the same section, the obtained results will be compared with each other and discussed in detail. The final consequences of the waveguide inhomogeneities are summarized in the section of “Conclusion”. The results of this detailed study show that despite the inhomogeneities are usually destructive, a group of their useful applications such as pulse reconstruction, pulse compression, and pulse shaping, can be proposed.

This paper presents distortion of pulses generated during different nonlinear three wave mixing processes caused by PPLN waveguide inhomogeneity and proposes a method to overcome it via induced upside down phase mismatch function. In this way, the inhomogeneity applied to the waveguide during construction and/or application can be estimated (not accurately measured). This helps to detect the inhomogeneity and fix it regardless of whether the inhomogeneity created along the waveguide is the result of environmental factors or was created during the construction of the waveguide. Up to our best knowledge, these results are novel and can pave new ways in the field of pulsed nonlinear optics and optical wavelength converters.

## Methods and results

In this section, nonlinear optical processes (SHG, DFG, and cSHG/DFG) used in wavelength converters are studied and the output results are compared with/without different types of waveguide inhomogeneities (constant, linear, quadratic, and upside-down quadratic).

### Second harmonic generation

Coupled fundamental mode is frequency doubled in a $\chi ^{(2)}$-nonlinear waveguide via SHG process. An efficient QPM-SHG in a uniform PPLN waveguide occurs when energy goes from fundamental mode to the SH one. However, the QPM condition can be affected by inhomogeneity of the PPLN waveguide. Then, energy transfer for SHG will not be efficient. To see the consequence of such an effect, pulsed SHG in PPLN waveguides with different types of inhomogeneities is studied first. The electric field of each interacting pulsed optical mode is defined as:

1 $$\begin{aligned} E_{k}\left( x,y,z,t\right) =A_{k}\left( x,t\right) E^{0}\left( y,z\right) \exp \left( j\left( \beta _k+j\frac{\alpha _k}{2}\right) x-\omega ^0_k t \right) \end{aligned}$$

with *y, z* as the transverse spatial dimensions, *x* as propagation direction, and *t* as the time of evolution. $A_k$ denotes the slowly varying amplitude (SVA) and $E^{0}$ is transverse distribution of normalized electric field of pulsed optical modes^20^. Also, $\beta _k$, $\alpha _k$ and $\omega ^0_k$ are the propagation constant of the modes, attenuation coefficient, and the central frequency of each pulse, respectively. Subscripts $k= f, sh$ stand for the fundamental and SH. Equation (2) shows the temporal profile of SVA for each interacting “$\textrm{sech}$” pulsed mode at the beginning of the PPLN waveguide (x = 0)^18^:

2 $$\begin{aligned} A_k\left( x=0, t\right) =\sqrt{P^0_k}\textrm{sech}\left( -\frac{1.76 t}{T_k^0}\right) , \end{aligned}$$

where, $P^0_k$ and $T_k^0$ represent maximum power and temporal full width at half maximum (FWHM) of each pulse, respectively. The nonlinear coupled mode equations describing the pulsed-SHG are:

3 $$ \begin{aligned} & \frac{\partial }{\partial x} A_{f}\left( x,t\right) + \beta_{p}^{\prime}\frac{\partial }{\partial t} A_{f}\left( x,t\right) +j\frac{\beta_{f}^{\prime\prime}}{2}\frac{\partial ^2}{\partial t^2} A_{f}\left( x,t\right) =j\kappa _{sh} A_{sh}\left( x,t\right) A_{f}^*\left( x,t\right) \exp \left( j\Delta \beta _{sh} x\right) ,\\&\frac{\partial }{\partial x} A_{sh}\left( x,t\right) + \beta _{sh}^{\prime}\frac{\partial }{\partial t} A_{sh}\left( x,t\right) +j\frac{\beta _{sh}^{\prime\prime}}{2}\frac{\partial ^2}{\partial t^2} A_{sh}\left( x,t\right) =j\kappa _{sh} A_{f}^2\left( x,t\right) \exp \left( - j\Delta \beta _{sh} x\right) , \end{aligned} $$

with $\kappa _{sh}$ as nonlinear coupling coefficient of the SHG process, $\beta '_k=\frac{\partial \beta }{\partial \omega }|_{\omega =\omega _k^0}$ and $\beta ''_k=\frac{\partial ^2 \beta }{\partial \omega ^2}|_{\omega =\omega _k^0}$ as inverse group velocity and the group velocity dispersion of pulses, respectively. $\Delta \beta _{sh}=2\beta _f-\beta _{sh}-\frac{2\pi }{\Lambda _{sh}}$ is the phase mismatch between fundamental and SH modes. $\Lambda _{sh}$ is the pitch of PPLN nonlinear grating for the SHG process. These parameters are listed in Table 1 according to Ref. ^18^.

**Table 1:** Parameters of the pulsed-SHG process in the PPLN optical waveguide according to Ref. ^18^.

Parameter	Value	Parameter	Value
$\lambda _f$	$1.55\,\,\upmu \hbox {m}$	$\kappa _{sh}$	$63\,\,\hbox {W}^{-1/2} /\hbox {m}$
$\lambda _{sh}$	$0.775\,\,\upmu \hbox {m}$	$\beta '_{f}$	$7.28\times 10^{-9}\,\,{\hbox {s/m}}$
$\beta ''_{f}$	$9.28\times 10^{-26}\,\,{\hbox {s}^{2}/\hbox {m}}$	$\beta '_{sh}$	$7.58 \times 10^{-9}\,\,{\hbox {s/m}}$
$\beta ''_{sh}$	$3.88\times 10^{-25}\,\,{\hbox {s}^{2}/\hbox {m}}$	$\Lambda _{sh}$	$18\,\,\upmu \hbox {m}$

When two pulses with the same $T_0$ temporal duration but different propagation constants of $\beta '_1$ and $\beta '_2$ propagate along an optical waveguide, the walk-off length ($L_{wo}$) between these two pluses is:

4 $$\begin{aligned} \begin{aligned} L_{wo}=\frac{T_{0}}{|\beta '_1-\beta '_2|} \end{aligned} . \end{aligned}$$

During this length ($L_{wo}$), co-propagating pulses have an amount of temporal overlap within desired nonlinear interaction can be occurred. It is worth mentioning that, for optical waveguides presented in this study, the overall evolution of interacting pulses is studied via their temporal tracing^11^.

**Figure 1:**
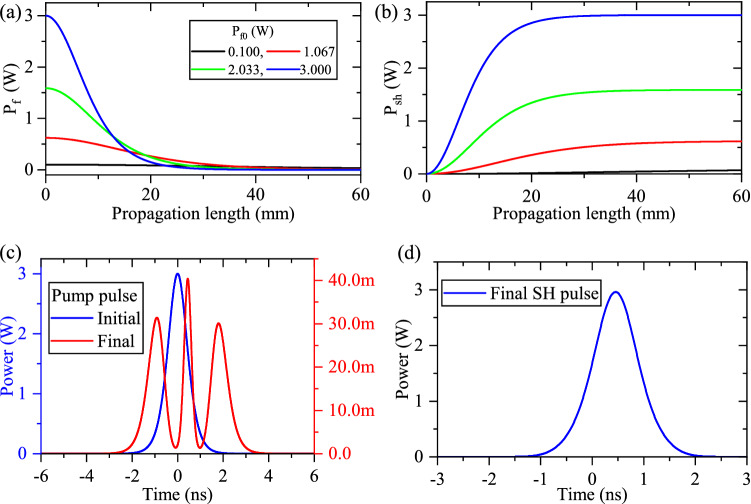
Optical power evolution of fundamental (**a**) and SH (**b**) modes with different values of coupled initial fundamental power, $P_{f0}=0.100,~1.067,~2.033,~3.00$ W, along a 60 mm homogeneous PPLN waveguide ($\Delta \beta =0$). For high input power, fundamental mode depletes in a shorter propagation length to generate the SH one. (**c**) Initial (blue- without depletion) and final (red- with depletion) shapes of fundamental pulse in the pulsed-SHG process for the same waveguide (L = 60 mm) and $P_{f0}=3.00~W$ peak power. Because of the power-dependent depletion of the fundamental mode, its final shape changes from the initial $\textrm{sech}^2$ one. (**d**) The final shape of the generated SH pulse. Since the generation rate of the SH mode is always positive, SH pulse maintains the $\textrm{sech}^2$ shape with a narrower FWHM.

Figure 1a,b illustrate the CW evolution of fundamental mode and SH one along a 60 mm homogeneous PPLN waveguide when different initial fundamental powers are considered ($P_{f0}$). With the gradual depletion of fundamental mode supplying the SH power in short propagation lengths, one can see that the depletion rate is independent of $P_{f0}$, whereas, for longer propagation lengths, fundamental modes with higher initial values deplete in shorter propagation lengths. When $P_{f0}$ is increased, SH-mode is created with higher generation rate. Hence, a continuous energy transfer from fundamental towards SH is evident in a homogeneous waveguide. Since the QPM condition is satisfied for SHG, even after the complete depletion of fundamental mode ($P_{f}=0$), no back conversion from SH to fundamental is observed. Thus, SHG is ideally a one-way process.

Furthermore, the initial (blue) and final (red) shape of the fundamental pulse are presented in Fig. 1c for the pulsed-SHG, when the coupled fundamental peak power is 3 W. These plots imply that the final fundamental pulse is not the same as the input $\textrm{sech}^2$ shape, while the final pulse shape of generated SH in Fig. 1d is kept in $\textrm{sech}^2$ shape. These phenomena can be justified using a simplified version of Eq. (3), which describe the CW-SHG under perfect-QPM condition in homogeneous waveguide ($\Delta \beta _{sh}=0$) as:

5 $$ \begin{aligned}&\frac{\partial }{\partial x} A_{f}\left( x,t\right) =j\kappa A_{sh}\left( x,t\right) A_{f}^*\left( x,t\right) ,\\&\frac{\partial }{\partial x} A_{sh}\left( x,t\right) =j\kappa A_{f}^2\left( x,t\right) . \end{aligned}$$

Since the second equation in equations 5 depends only on $A_{f}$, when fundamental mode depletes completely ($A_{f}=0$), the right-hand sides of the coupled equations become zero and the nonlinear process ends. Therefore no back conversion can be observed. According to this proof about the evolution of fundamental power in CW-regime, in case of long propagation lengths, the temporal components of fundamental pulse with higher amplitudes deplete more^11^. This leads to the distortion of the output fundamental pulse (as in Fig. 1c). In addition, due to the always monotonic generation rate of the SH mode in homogeneous waveguide ($\Delta \beta _{sh}=0$), the shape of output SH pulse is almost $\textrm{sech}^2$ with a narrower FWHM (as in Fig. 1d).

**Figure 2:**
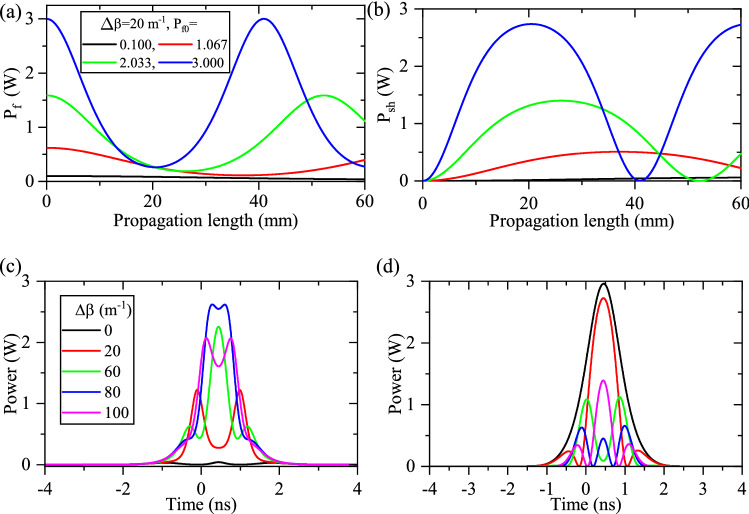
Power evolution of fundamental (**a**) and SH (**b**) modes in a waveguide (L = 60 mm) with constant phase mismatch $\Delta \beta =20\,\hbox {m}^{-1}$, when different coupled fundamental powers ($P_{f0}=0.100,~1.067,~2.033,~3.00$ W) are considered. The output pulse shape of fundamental (**c**) and SH (**d**) modes with different values of constant phase mismatch ($\Delta \beta =0,~20,~60,~80$, and ${100}\,{{\text {m}}^{-1}}$). Because of the periodic depletion of fundamental and SH powers, their final shapes are changed from $\textrm{sech}^2$ one, more drastically.

In contrast to the above description, any inhomogeneity of waveguide formation and/or QPM grating results in imperfect phase matching during the nonlinear process. This causes the pulse deformation of the interacting modes. Thus, with $\Delta \beta \ne 0$ and $\Delta \beta =$constant, the routine energy transfer from fundamental to SH pulses can not be established and periodic energy exchange between them is expected. Here, different types of waveguide inhomogeneities are induced and their effects on the SHG process are studied. Figure 2a,b show the calculated power evolution of fundamental and SH modes, respectively when constant inhomogeneity equivalent to $\Delta \beta =20\,\hbox {m}^{-1}$ is induced. The coupled fundamental powers are the same as what is presented in Fig. 1. Comparing Figs. 2b and 1b, it can be deduced that under the phase mismatched process, the order of reaching different fundamental and SH components to the end of the waveguide disappears partially. This leads to some distortions in the final shape of the SH pulse.

Figure 2c,d depict the output pulse shapes of fundamental and SH pulses when constant inhomogeneities equivalent to phase mismatches $\Delta \beta =0,~20,~60,~80$, ${100}\,{{\text {m}}^{-1}}$ are induced. As expected, inducing the constant phase mismatch in the PPLN waveguide leads to stronger distortion in the final shape of fundamental and SH pulses. For higher values of $\Delta \beta$, transferring energy from fundamental pulse into SH one is reduced. Therefore, the fundamental pulse is not negligible at the end of the waveguide. When $\Delta \beta$ increases, the shape of both fundamental and SH pulses is changed from the $\textrm{sech}^2$ one. The presented results in this section show that the constant imperfect PPLN waveguide leads to the reduction in the conversion efficiency on the one hand, and distortion of the output SH pulse shape on the other hand.

**Figure 3:**
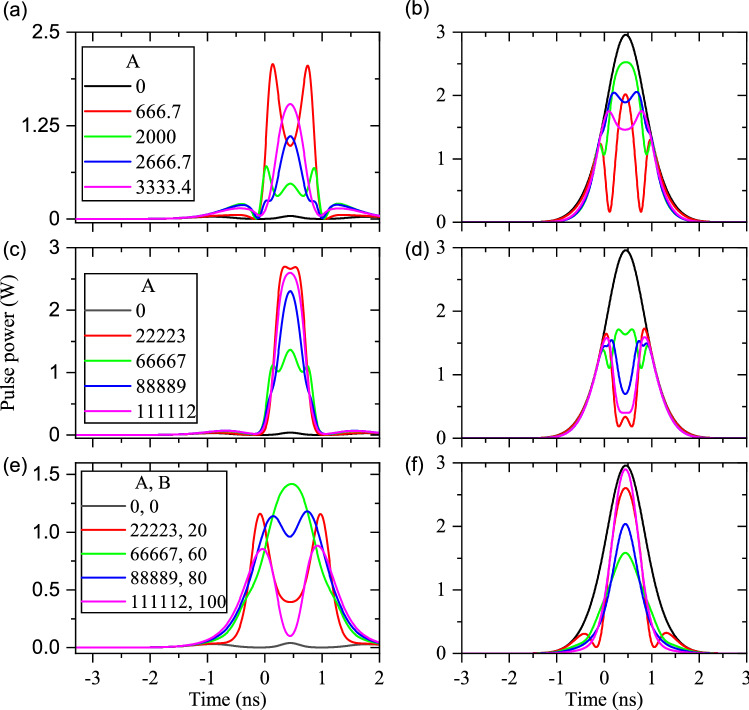
Effects of different types of linear waveguide (L = 60 mm) inhomogeneities for the pulsed-SHG process. (**a**) and (**b**) are the fundamental and SH pulse shapes under the effects of linear inmomogeneity ($\Delta \beta = A\left( x-0.03\right)$), respectively. (**c**) and (**d**) display the fundamental and SH pulse shapes, respectively, when $\Delta \beta$ changes quadratically ($\Delta \beta = A\left( x-0.03\right) ^2$). (**e**) and (**f**) represent the fundamental and SH pulse shapes, respectively, when $\Delta \beta$ changes upside-down quadratically ($\Delta \beta = -A\left( x-0.03\right) ^2+B$).

In addition, the combination of a constant phase mismatch with a linear one is considered, $\Delta \beta = A (x-0.03)$, where *A* is constant and chosen so that $\Delta \beta$ becomes 0, 20, 60, 80, ${100}\,{{\text {m}}^{-1}}$ at the center of the waveguide. Figure 3a,b show the effects of linear $\Delta \beta$ on the calculated output fundamental and SH pulses (L = 60 mm), respectively. As it can be seen, linear $\Delta \beta$ leads to the reduction of SHG efficiency and a substantial distortion of the output pulse shapes, simultaneously. Moreover, the quadratic PPLN waveguide inhomogeneity (i.e $\Delta \beta =A (x-0.03)^2$) is defined so that the minimum of $\Delta \beta$ is at the center of the waveguide (L = 30 mm) and it grows quadratically on both sides (values of *A* are chosen so that the maximum of $\Delta \beta$ becomes 0, 20, 60, 80, ${100}\,{{\text {m}}^{-1}}$ at both ends). The out-coupled pulses are depicted in Fig. 3c,d. As was expected, the overall SHG conversion efficiency is reduced with *A* and the fundamental pulse becomes stronger at the end of the waveguide. According to Fig. 3d, higher values of quadratic variations of $\Delta \beta$ change the SH pulse shape in such a way that the output SH mode nearly splits into two pulses. Therefore, in the quadratic variations of $\Delta \beta$, the central components of the SH mode in the CW picture deplete more rapidly with respect to the outer ones.

To complete the discussion in this subsection, the upside-down quadratic function of $\Delta \beta$ (i.e $\Delta \beta =-A (x-0.03)^2+B$ ) is induced, so that $\Delta \beta$ reaches its maximum at the central part of the waveguide (L = 30 mm) and decreases quadratically on both sides. The relevant values of *A* and *B* are reported in Fig. 3e-inset and are chosen in such a way that $\Delta \beta$ becomes 0, 20, 60, 80, and ${100}\,{{\text {m}}^{-1}}$ at the center of the waveguide. The effects of the upside-down quadratic function of $\Delta \beta$ on the final shapes of fundamental and SH pulses are presented in Fig. 3e,f, respectively. The results show that fundamental pulse shapes are distorted while $\Delta \beta$ varies upside-down quadratically. However, as can be seen in Fig. 3f, the final shape of SH pulse starts reconstructing itself with a narrower FWHM (see the magenta plot in Fig. 3f). Therefore, if a suitable upside-down quadratic $\Delta \beta$ is intentionally induced in the PPLN waveguide, it is possible to compress the final SH pulse, too.

It is helpful mentioning that, any return of energy from SH pulse in fundamental one (SH pulse distortion), could be a sign of waveguide inhomogeneity. Thus, this can be used as a fast laboratory method to survey the precision of PPLN waveguide fabrication. In the rest of this section, the same linear, quadratic and upside-down quadratic $\Delta \beta$ functions (equivalent to different waveguide inhomogeneities) are used to investigate their effects on DFG and cSHG/DFG processes.

### Difference frequency generation

A nonlinear optical process in which an the incoming signal wave ($\omega _s$) interacts with a strong pump wave ($\omega _p$) to generate a new idler wave ($\omega _i$) is known as DFG ($\omega _i=\omega _p-\omega _s$). In the presence of the generated idler wave, an additional signal wave can be generated leading to amplification of incoming signal wave known as optical parametric amplification (OPA). Since this process is used for wavelength conversion and amplification in an optical telecommunication system, it is crucial to have a thorough vision of the waveguide inhomogeneitiesthe in the pulsed regime of interaction. The coupled mode equations describing the pulsed DFG interaction are written in equations (6);

6 $$ \begin{aligned} & \frac{\partial }{\partial x} A_{p}\left( x,t\right) + \beta _p'\frac{\partial }{\partial t} A_{p}\left( x,t\right) +j\frac{\beta _p''}{2}\frac{\partial ^2}{\partial t^2} A_{p}\left( x,t\right) =j\frac{\omega _p}{\omega _i}\kappa A_{s}\left( x,t\right) A_{i}\left( x,t\right) \exp \left( j\Delta \beta _{df} x\right) ,\\&\frac{\partial }{\partial x} A_{s}\left( x,t\right) + \beta _{s}'\frac{\partial }{\partial t} A_{s}\left( x,t\right) +j\frac{\beta _{s}''}{2}\frac{\partial ^2}{\partial t^2} A_{s}\left( x,t\right) =j\frac{\omega _s}{\omega _i}\kappa A_{p}\left( x,t\right) A_{i}^* \left( x,t\right) \exp \left( - j\Delta \beta _{df} x\right) ,\\&\frac{\partial }{\partial x} A_{i}\left( x,t\right) + \beta _{i}'\frac{\partial }{\partial t} A_{i}\left( x,t\right) +j\frac{\beta _{i}''}{2}\frac{\partial ^2}{\partial t^2} A_{i}\left( x,t\right) =j\kappa A_{p}\left( x,t\right) A_{s}^* \left( x,t\right) \exp \left( - j\Delta \beta _{df} x\right) . \end{aligned} $$

The subscripts *p*, *s*, and *i* stand for the pump, signal, and idler, respectively. $\Delta \beta _{df}=\beta _s+\beta _i-\beta _{p}-\frac{2\pi }{\Lambda _{df}}$ denotes the total phase mismatch which is zero for the perfect QPM condition (homogeneous waveguide) and non-zero for inhomogeneous waveguide, while $\Lambda _{df}$ is the pitch of nonlinear PPLN grating for DFG interaction. To be specific, $\lambda _p=775\,\hbox {nm}$, $\lambda _s=1560\,\hbox {nm}$ and $\lambda _i=1540\,\hbox {nm}$ are considered. In this way, the coupling coefficient is the same as that of the SHG process^18^.

**Figure 4:**
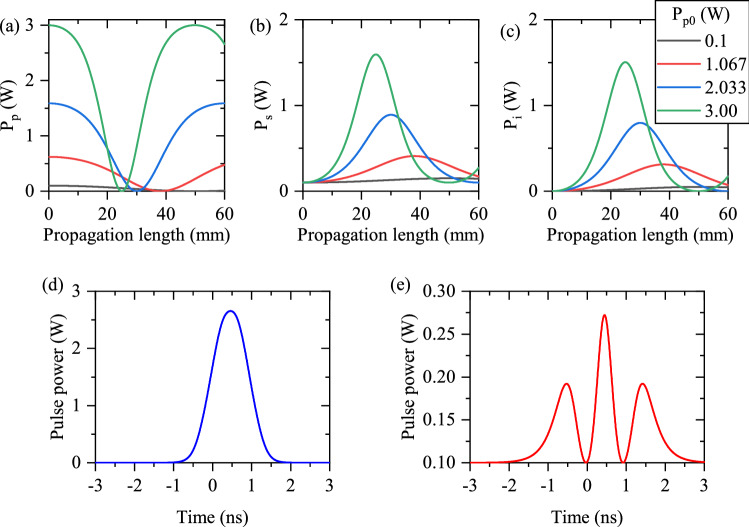
Propagation of different components of pump (**a**), signal (**b**), and idler (**c**) modes along a $60\,\hbox {mm}$ PPLN waveguide for the CW-DFG process when $\Delta \beta =0$ (homogeneous waveguide) is satisfied, with different values of $P_{p0}=0.1,~1.067,~2.033,~3.00~W$. Out-coupled pump (**d**) and signal (**e**) pulses.

Since the study on the CW picture of the DFG interaction clarifies the formation of pump, signal, and idler pulses, this subsection is started with the examination of the CW-DFG process under the perfect QPM circumstance. Figure 4a–c illustrate the power evolution of the pump, amplified signal, and generated idler, respectively. The PPLN waveguide is homogeneous ($\Delta \beta _{df}=0$) and coupled pump powers $P_{p0}$ vary while the input signal power $P_{ps0}$ is $0.1\,\hbox {w}$. The results show gradual depletion of the pump along the PPLN waveguide. For those components whose $P_{p0}$ are higher, the depletion rates are stronger. But, when the pump mode depletes completely, the amplified signal and the generated idler (see Fig. 4b,c) give their energies to the pump through the nonlinear sum frequency generation process; thus, the pump is regenerated. Although there is a higher rate of depletion for stronger pump components, but after the regeneration of pump mode, the pump components are generated regularly. Therefore, it is expected that the output shape of the pump pulse is not distorted (as in panel (d) of Fig. 4). As one can see, the order of their different components changes at the end of the waveguide (L = 60 mm). Thus, the distortion in the final shapes of signal and idler pulses is expected. This effect is presented in Fig. 4e, where the final shape of the signal pulse is distorted due to the mentioned irregular variation of different signal components.

**Figure 5:**
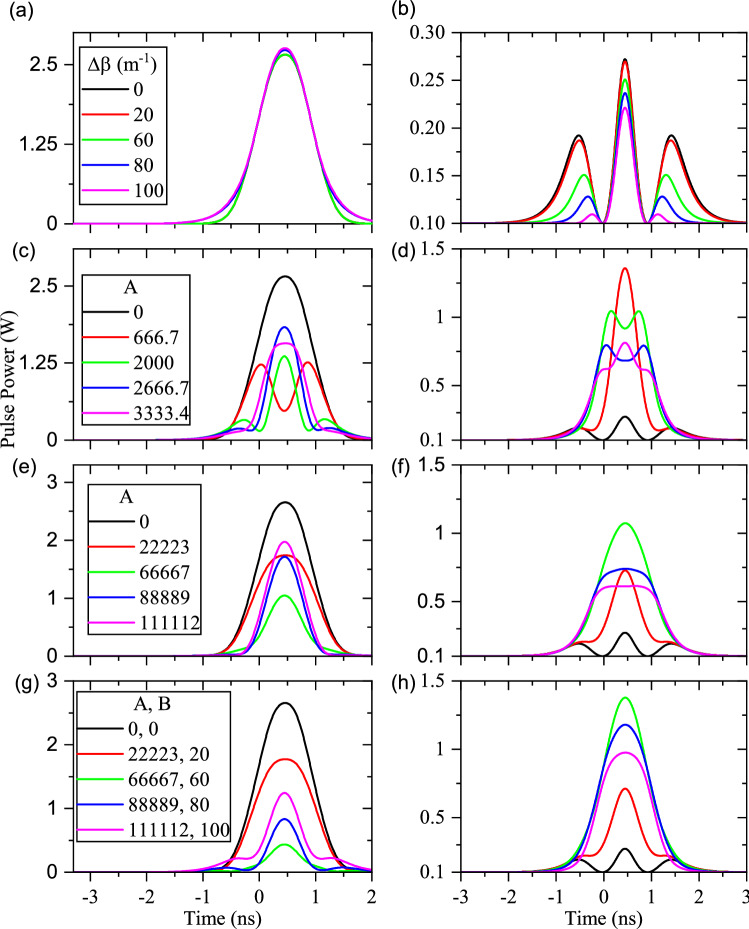
Effects of different types of waveguide inhomogeneity for the pulsed-DFG process. (**a**) and (**b**) depict the pump and signal/idler pulse shapes under the effects of constant inhomogeneity ($\Delta \beta = 0,~20,~60,~80$, and ${100}\,{{\text {m}}^{-1}}$), respectively. (**c**) and (**d**) respectively, are the pump and signal/idler pulse shapes, under the effects of linear inhomogeneity ($\Delta \beta = A\left( x-0.03\right)$). (**e**) and (**f**) are the pump and signal/idler pulse shapes, respectively, when $\Delta \beta$ changes quadratically ($\Delta \beta = A\left( x-0.03\right) ^2$). (**g**) and (**h**) represent the pump and signal/idler pulse shapes, respectively, when $\Delta \beta$ changes upside-down quadratically ($\Delta \beta = -A\left( x-0.03\right) ^2+B$).

The effects of constant waveguide inhomogeneity are studied in Fig. 5a,b for the pump and signal/idler, respectively. Because of the similar behavior of signal and idler pulses, the final shape of the signal pulse is presented only. The result shows that the constant inhomogeneity has small influence on the final shape of the pump pulse, but it considerably changes the final shape of signal/idler pulses. The side lobes of the signal/idler pulses are faded out by increasing the constant $\Delta \beta$ (for example if $\Delta \beta =100\,{\text {m}}^{-1}$). Figure 5c,d display the resultant shapes of output pump and signal/idler pulses, for linear $\Delta \beta$, respectively. Such a $\Delta \beta$ leads to the distortion of final pump pulse. Further, it is possible to have cases where the shape of signal/idler pulses can be regenerated to some extent (the red plot in Fig. 5d). However, finding the proper parameter of the linear $\Delta \beta$ needs precise and exact laboratory settings.

The results of quadratic variations of $\Delta \beta$ are also illustrated in panel (e) and (f) of Fig. 5. Finally, the effects of upside-down quadratic waveguide inhomogeneity on the resultant shapes of the pump and signal/idler pulses are plotted in Fig. 5g,h, too. It can be concluded that the pump pulse shapes are distorted from the $\textrm{sech}^2$ one. However, it is possible to reconstruct and amplify the $\textrm{sech}^2$-like shape for the signal/idler pulses using suitable upside-down quadratic incomplete QPM PPLN gratings. The importance of this process becomes clear when $\Delta \beta$ is zero, but the final shapes of the signal/idler pulses are not $\textrm{sech}^2$ at all (see the black line in Fig. 5h).

### cSHG/DFG

In a DFG process, the frequency of the pump mode is approximately two times of the frequencies for signal and idler modes. This yields in the excitation of higher order spatial modes for the pump frequency in optical waveguides ^9^ which are mono-mode for signal and idler. This unwanted excitation of the higher order modes degrades the efficient operation of the waveguide-based DFG process. Such an unwanted mode excitation can be suppressed by cascading the generation of second harmonic and difference frequency (as cSHG/DFG)^9,11^. In the cSHG/DFG process, the needed pump of DFG can be itself generated, mode selectively, in the waveguide via SHG process. The generated SH mode interacts with the signal mode to generate the idler one. Such as the normal DFG process, the signal mode experiences a parametric gain. Interestingly, the QPM condition of the SHG process is in the same band as the DFG one. Since cSHG/DFG involves SHG and DFG interactions, any waveguide inhomogeneity has a strong influence on the output signal/idler pulses. Equation (7) represent the pulsed cSHG/DFG process:

7 $$ \begin{aligned}  & \frac{\partial }{\partial x} A_{p}\left( x,t\right) + \beta _p'\frac{\partial }{\partial t} A_{p}\left( x,t\right) +j\frac{\beta _p''}{2}\frac{\partial ^2}{\partial t^2} A_{p}\left( x,t\right) = j\kappa A_{sh}\left( x,t\right) A_{p}^*\left( x,t\right) ,\\&\frac{\partial }{\partial x} A_{sh}\left( x,t\right) + \beta _{sh}'\frac{\partial }{\partial t} A_{sh}\left( x,t\right) +j\frac{\beta _{sh}''}{2}\frac{\partial ^2}{\partial t^2} A_{sh}\left( x,t\right) = j\kappa A_{p}^2\left( x,t\right) +j\frac{\omega _{sh}}{\omega _i}\kappa A_{s}\left( x,t\right) A_{i} \left( x,t\right) \exp \left( j\Delta \beta _{df} x\right) ,\\&\frac{\partial }{\partial x} A_{s}\left( x,t\right) + \beta _{s}'\frac{\partial }{\partial t} A_{s}\left( x,t\right) +j\frac{\beta _{s}''}{2}\frac{\partial ^2}{\partial t^2} A_{s}\left( x,t\right) = j\frac{\omega _s}{\omega _i}\kappa A_{sh}\left( x,t\right) A_{i}^* \left( x,t\right) \exp \left( - j\Delta \beta _{df} x\right) ,\\&\frac{\partial }{\partial x} A_{i}\left( x,t\right) + \beta _{i}'\frac{\partial }{\partial t} A_{i}\left( x,t\right) +j\frac{\beta _{i}''}{2}\frac{\partial ^2}{\partial t^2} A_{i}\left( x,t\right) = j\frac{\omega _s}{\omega _i}\kappa A_{sh}\left( x,t\right) A_{s}^* \left( x,t\right) \exp \left( - j\Delta \beta _{df} x\right) . \end{aligned}$$

**Figure 6:**
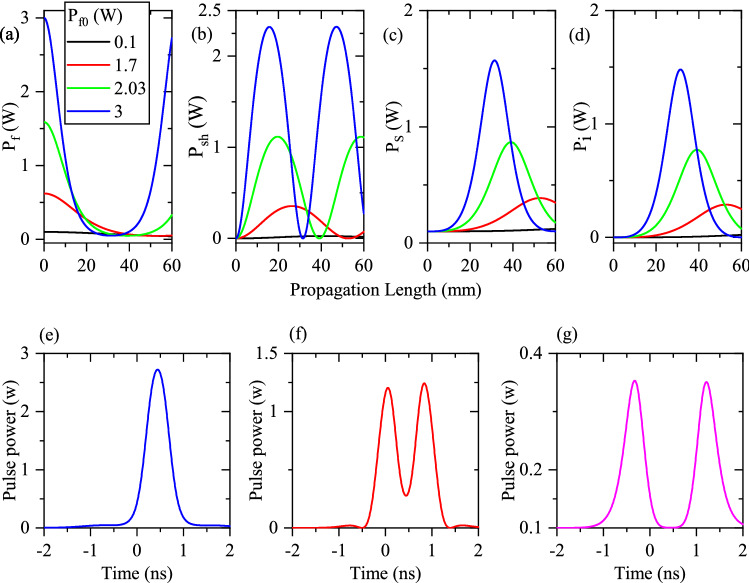
Propagation of different components of fundamental (**a**), generated SH (**b**), signal (**c**), and idler (**d**) modes with different values of $P_{f0}=0.1,~1.7,~2.03,~3.00~ W$ in a 60 mm long PPLN waveguide for the CW-cSHG/DFG process when $\Delta \beta =0$ (homogeneous waveguide). The out-coupled fundamental (**e**), generated SH (**f**), and signal (**g**) pulses.

Again, CW-based cSHG/DFG interaction clarifies the behavior of different power components for fundamental, SH, signal, and idler pulses. Figure 6a shows the propagation of fundamental mode for its different input values ($P_{f0}$), for the CW regime in a homogeneous waveguide. Its regular depletion is evident. Here, instead of normal SHG, the regeneration of fundamental mode can be observed after its complete depletion, which is due to the presence of signal and idler modes. The expected $\textrm{sech}^2$ shape is for output fundamental pulse, as displayed in panels (e) of Fig. 6. Generated SH mode for different values of $P_{f0}$ is depicted in Fig. 6b. The results show that the SH mode of higher fundamental powers $P_{f0}$ depletes faster to generate signal and idler modes. Therefore, a central dip appears in the resultant SH pulse which is illustrated in Fig. 6f. The resultant signal and idler modes are respectit isively displayed in the panels (c) and (d) of Fig. 6. Due to the regeneration of fundamental mode, the signal/idler modes deplete completely at the end of the waveguide for higher $P_{f0}$ (see Fig. 6c,d). Such an effect leads to the splitting of resultant out-coupled signal/idler pulses (see Fig. 6g). Therefore, it is concluded that the peak power of fundamental pulse and the waveguide length should be chosen exactly to prevent the splitting of the final signal/idler pulse at the output end of the waveguide.

**Figure 7:**
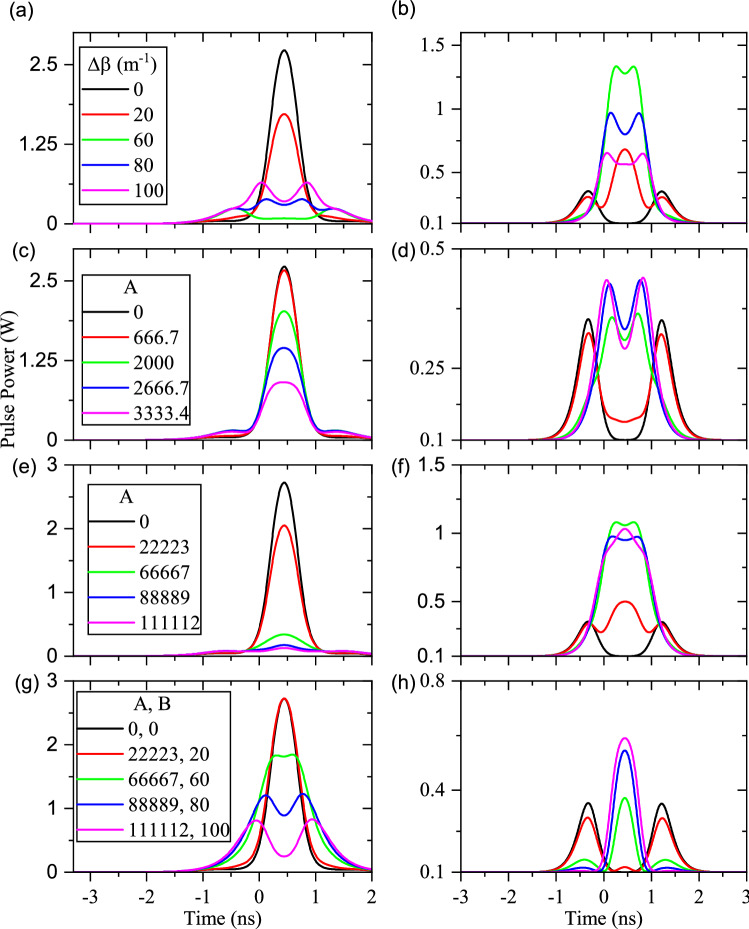
Out-coupled pulses during the cSHG/DFG process when different types of waveguide inhomogeneities are assumed (L = 60 mm). (**a**) and (**b**) depict respectively the output fundamental and signal/idler pulse, with constant waveguide inhomogeneity ($\Delta \beta = 0, 20, 60, 80$ and ${100}\,{{\text {m}}^{-1}}$). (**c**) and (**d**) are the output fundamental and signal/idler pulse, respectively, when a linear waveguide inhomogeneity is applied ($\Delta \beta = A\left( x-0.03\right)$). (**e**) and (**f**) respectively illustrate the output fundamental and signal/idler pulse, when quadratic changes of $\Delta \beta$ are considered ($\Delta \beta = A\left( x-0.03\right) ^2$). (**g**) and (**h**) represent the output fundamental and signal/idler pulse shapes, respectively, when the changes of $\Delta \beta$ are quadratic upside-down ($\Delta \beta = -A\left( x-0.03\right) ^2+B$).

The effects of constant, linear, quadratic, and upside-down quadratic types of waveguide inhomogeneity are illustrated in the ordered panels of Fig. 7, respectively. To be specific, $\lambda _f=1550\,\hbox {nm}$ and $\lambda _s=1540\,\hbox {nm}$ are used for calculations. Panel (a) and (b) of Fig. 7 show the effect of constant $\Delta \beta$ on the final shapes of fundamental and signal/idler pulses at the output end of the waveguide. Results indicate that the constant waveguide inhomogeneity leads to distortions of the final fundamental, signal, and idler pulses. Effects of linear waveguide inhomogeneity (equivalent to a linear variation of $\Delta \beta$) are depicted in Fig. 7c,d for output fundamental and signal/idler pulses, respectively. Although the linear inhomogeneity does not change the final fundamental pulse considerably, but it meaningfully distorts the final signal/idler pulse shapes. Moreover, the quadratic inhomogeneities of (variations $\Delta \beta$ quadratically) lead to the distortions of final shapes of the fundamental and signal/idler pulses (see Fig. 7e,f).The upside-down quadratic variations of $\Delta \beta$ result in the depletion and strong distortion of fundamental pulses, as depicted in Fig. 7g. The $\textrm{sech}^2$ shape of the signal/idler pulse at the end of the waveguide can be regenerated using a suitable upside-down quadratic variations of $\Delta \beta$ (see the magenta plot in Fig. 7h). Since the cSHG/DFG process aims to convert the signal pulses to the wavelength shifted idler ones, undistorted shape of generated signal pulses (=idler) are important. Therefore, the wavelength conversion of short data pulses becomes feasible by using the cSHG/DFG process and suitable upside-down quadratic variations of $\Delta \beta$.

## Conclusions

High-capacity optical communication networks based on wavelength division multiplexing are rapidly developing. In such networks, data signals in different wavelength channels are short pulses where their amplitude, phase, time, and/or polarization are modulated in advanced formats. Therefore, addressing each channel needs an unoccupied wavelength-route in each node. All-optical wavelength converters can change the wavelength of any data channel to the desired one, in which a free out-route channel is available in the node. Thus, the noiseless wavelength converters that can be operated in short pulse regime are essential tools to overcome contention resolution in modern communication networks. Beside various types of traditional wavelength converters, $\chi ^2$-type integrated nonlinear optical ones are more suitable to build a compact low-loss all-optical node. In such a waveguide-based device, any imperfect functionalities can be addressed to the inhomogeneity of the waveguide. This paper presents the detailed study of out-coupled wavelength converted pulses affected by various types of inhomogeneities induced in a PPLN straight waveguide. The nonlinear SHG, DFG, and cSHG/DFG processes are numerically studied to show that any constant, linear, and quadratic waveguide inhomogeneities cause short pulse (down to 1 ns) distortion in the nonlinear optical wavelength converters. It is also shown that by suitable induced upside-down inhomogeneities, one can compensate the device fabrication/operation faults. The important results of this study are summarized below:

1. The SHG is a one-way process, in which fundamental transmits its energy into the SH pulse in a homogeneous waveguide. Any return of energy into the pump mode, which results in pulse distortion, could be a sign of waveguide inhomogeneity. This fact can be used as a fast laboratory test of waveguide homogeneity. The obtained results also show that using a deliberately applied upside-down quadratic variation of inhomogeneity regenerates the $\textrm{sech}^2$ pattern with a narrow SH pulse. Pulse narrowing^21,22^ is very crucial in high-speed modulation for dense WDM networks.
2. The resultant signal/idler pulse shape in the DFG process could be deformed even in an ideal PPLN optical waveguide. Here, the induced waveguide inhomogeneity can be purposely utilized to reconstruct the $\textrm{sech}^2$ pattern. According to the results presented in this paper, upside-down quadratic inhomogeneity leads to reconstruction of pulse shape, in addition to further signal parametric gain.
3. In the cSHG/DFG process, the final shape of signal/idler pulses are changed from the $\textrm{sech}^2$ pattern, depending on the length of waveguide. Here again, using the upside-down quadratic variation leads to an almost $\textrm{sech}^2$ shape.

Furthermore, other different types of waveguide inhomogeneities can be thought as the superposition of obtained results. Therefore, their effects on the final shape of resultant pulses can be collected in a database. The presented simulation results show that even if the output pulses of a nonlinear process in the presence of two different inhomogeneities are somewhat similar and have little difference from each other, they will have a big difference in the other two nonlinear processes. In addition, efficiency of each NLO process gives a direct consequence of the duty cycle of the PPLN grating. This is another degree of freedom to factor out the inhomogeneity caused by grating and obtained the inhomogeneities of the LN waveguide only. Therfore, creating three databases based on three nonlinear processes SHG, DFG and cSHG/DFG, together with duty cycle measurements of the PPLN gratings, enable the machine learning algorithms to detect the inhomogeneities of the waveguide.

## Data Availability

There is no additional dataset for this study. All data generated or analyzed during this study are included or referred in this published article. However, For the clarification and convenience, any needed datasets used and/or analyzed during the current study is available from the corresponding author on reasonable request.
